# Molecular epidemiology and pathogenicity of H5N1 and H9N2 avian influenza viruses in clinically affected chickens on farms in Bangladesh

**DOI:** 10.1080/22221751.2021.2004865

**Published:** 2021-11-29

**Authors:** Ripatun Nahar Ripa, Joshua E. Sealy, Jayna Raghwani, Tridip Das, Himel Barua, Md. Masuduzzaman, A. K. M. Saifuddin, Md. Reajul Huq, Mohammad Inkeyas Uddin, Munir Iqbal, Ian Brown, Nicola S. Lewis, Dirk Pfeiffer, Guillaume Fournie, Paritosh Kumar Biswas

**Affiliations:** a Department of Microbiology and Veterinary Public Health, Chattogram Veterinary and Animal Sciences University, Chattogram, Bangladesh; b Avian Influenza Viruses Group, The Pirbright Institute, Pirbright, UK; c University of Oxford, Oxford, UK; d Poultry Research and Training Centre, Chattogram Veterinary and Animal Sciences University, Chattogram, Bangladesh; e Department of Pathology and Parasitology, Chattogram Veterinary and Animal Sciences University, Chattogram, Bangladesh; f Department of Physiology, Biochemistry and Pharmacology, Chattogram Veterinary and Animal Sciences University, Chattogram, Bangladesh; g Department of Livestock Services, District Livestock Office, Chattogram, Bangladesh; h The Royal Veterinary College, Hatfield, UK; i Animal and Plant Health Agency, Addlestone, UK; j Jockey Club College of Veterinary Medicine and Life Sciences, City University of Hong Kong, Hong Kong, People’s Republic of China

**Keywords:** Avian influenza, virus evolution, phylodynamics, co-infection, antigenicity, H5N1, H9N2

## Abstract

Avian influenza virus (AIV) subtypes H5N1 and H9N2 co-circulate in poultry in Bangladesh, causing significant bird morbidity and mortality. Despite their importance to the poultry value chain, the role of farms in spreading and maintaining AIV infections remains poorly understood in most disease-endemic settings. To address this crucial gap, we conducted a cross-sectional study between 2017 and 2019 in the Chattogram Division of Bangladesh in clinically affected and dead chickens in farms with suspected AIV infection. Viral prevalence of each subtype was approximately 10% among farms for which veterinary advice was sought, indicating high levels of virus circulation in chicken farms despite the low number of reported outbreaks. Co-circulation of both subtypes was common in farms, with our findings suggest that in the field, the co-circulation of H5N1 and H9N2 can modulate disease severity, which could facilitate an underestimated level of AIV transmission in the poultry value chain. Finally, using newly generated whole-genome sequences, we investigate the evolutionary history of a small subset of H5N1 and H9N2 viruses. Our analyses revealed that for both subtypes, the sampled viruses were genetically most closely related to other viruses isolated in Bangladesh and represented multiple independent incursions. However, due to lack of longitudinal surveillance in this region, it is difficult to ascertain whether these viruses emerged from endemic strains circulating in Bangladesh or from neighbouring countries. We also show that amino acids at putative antigenic residues underwent a distinct replacement during 2012 which coincides with the use of H5N1 vaccines.

## Introduction

Low and highly pathogenic avian influenza viruses (LPAIVs and HPAIVs, respectively) are endemic to many regions of Africa and Asia [1–4]. The highly pathogenic avian influenza (HPAI) H5N1 A/goose/Guangdong/1996 lineage (Gs/GD) of H5 viruses are a major lineage that has evolved and diversified over the past ∼25 years with descendant viruses making up distinct and complex clades. Contemporary H5 viruses circulate in aquatic and terrestrial poultry and periodically transmit to wild birds which facilitates enhanced geographical dispersion and extended virus reassortment and evolution [5,6]. While HPAIVs (e.g. H5N1 hereafter referred to as H5) are associated with high mortality in poultry, LPAIVs (e.g. H9N2) typically cause mild or subclinical disease. However, co-infection of LPAIVs with other viruses or bacteria can cause severe morbidity and mortality [7,8]. Besides posing a threat to poultry health and food security, H5 and H9N2 viruses also sporadically infect humans [9,10]. Their potential to mutate and reassort drives viral fitness in avian hosts but also perpetuates a risk for new variants to emerge that are capable of zoonotic and human-to-human infection [11,12]. Therefore, surveillance for AIVs is important to characterize the genetic and phenotypic properties of viruses and to assess the potential impact on both food systems and putative zoonotic risk. Such monitoring is also required to assess virus dissemination, establish hotspots of viral diversity, and inform the design of risk-based surveillance and control programmes.

Live bird markets (LBMs) are an essential component of the poultry value chain where H5 and H9 viruses are frequently detected [13–15]. In contrast, detection of AIVs on farms is rare. This observation likely reflects low viral prevalence in farms [16] and underreporting of disease outbreaks by farmers due to limited access to veterinary services and concerns about the financial consequences of confirming HPAIV on their premises. Surveillance activities predominantly target LBMs where poultry from different species, breed, and geographical origins intermingle. This approach is cost-effective as it can assess the virus genetic diversity circulating in chicken populations supplied to LBMs from a variety of sources. However, this surveillance strategy suffers from limitations. First, it is difficult to ascertain the source location of the viruses detected in the markets. As a result, we have limited insight into how viruses disseminate at relevant spatiotemporal scales. Second, the complex viral transmission dynamics along the trading networks suggests that the virus composition could markedly differ between market and farms. Specifically, it is unclear if lineages observed in farming systems are absent or present at a low prevalence in the markets. Third, unless AIV infections are detected in farms, it is difficult to assess their associated mortality under field conditions. Therefore, surveillance and genomic sequencing of H5 and H9 viruses in farmed chickens is critical to improving our understanding of AIV diversity and dissemination in endemically affected regions.

To address this gap, we investigated the molecular epidemiology and pathogenicity of H5N1 and H9N2 viruses isolated from farmed chickens in Chattogram division, Bangladesh. Samples were collected as part of a cross-sectional study between October 2017 and April 2019, from clinically sick or dead birds submitted by commercial farmers to two veterinary clinicians and a veterinary diagnostic laboratory in Chattogram. Our findings revealed a high co-occurrence of H5 and H9 viruses in clinically affected farms for which veterinary advice was sought, and we found this to be indicative of an underappreciated level of AIV circulation in farms, possibly due to reduced chicken mortality compared to farms with H5N1 alone. Furthermore, whole-genome sequences from four H5N1 and seven H9N2 viruses indicate multiple independent viral incursions over the study period, which likely emerged from endemic lineages circulating in Bangladesh. Lastly, analysis of genetic markers suggests there is notable selective pressure acting on H5N1 viruses, which has led to the apparent fixation of amino acids at putative antigenic residues that coincides with the introduction of H5N1 vaccines in Bangladesh in 2012.

## Materials and methods

### Ethical statement

This study was approved by the Ethics Committee (EC) of the Chattogram Veterinary and Animal Sciences University under the Memo no. CVASU/Dir (R&E)EC/2019/126/(8).

### Sample collection

The two veterinarians and the Animal Disease Diagnostic Laboratory (ADDL) hosted at the Poultry Research and Training Centre (PRTC) were based at the Chattogram Veterinary and Animal Sciences University (CVASU). A total of 262 farms were recruited, allowing the estimation of a prevalence of infection of 10% among clinically affected chicken flocks, with a precision of 4% and a confidence level of 95%. For each farm, two to five sick and/or dead chickens were sampled, depending on the number of birds supplied. Oropharyngeal swabs from sick chickens, and tracheal or oropharyngeal swabs from dead chickens were collected in a falcon tube containing 3 mL of a virological transport medium (VTM). The VTM was prepared locally, following the Standard Operating Procedure (Ref. BPU 1551; Implementation date 27/11/2013) of the UK Animal and Plant Agency, with penicillin G being replaced with benzathine penicillin. After collection, samples were stored at −80°C until laboratory investigations. The farm owner or their representative was asked about the flock size, management practices and mortality rate in the week preceding the sample collection using a structured questionnaire.

### Viral RNA extraction and RT-PCR

MagMax-96 Viral RNA Isolation Kit (Ambion life technologiesTM) or QIAamp Viral RNA Mini Kit (QiagenTM) was used to extract viral RNA from the swab samples stored in VTM following the manufacturer’s instructions. The extracted RNAs were preserved at −80°C until further investigations. The CSIRO (The Commonwealth Scientific and Industrial Research Organization) (www.csiro.au) Australian Animal Health Laboratory protocols were used to detect the presence of the M (matrix) gene of AIV, and subsequently, the presence of H5 and H9 in the samples tested positive for the M gene, by real-time reverse transcription polymerase chain reaction (rRT-PCR) assays [17]. AgPath–ID One-Step RT-PCR kit for 100 reactions (ThemoFisher Scientific) was used for all these assays. An rRT-PCR test for any of the genes was done with 25 µL volume. The sequences of primers and probes are shown in Table S2. The test assays were run on an AB 7500 Fast Real-Time PCR System (Applied BiosystemTM). A Ct (cycle threshold) of less than 40 with a characteristic amplification curve was considered as a positive result for any of the genes tested.

### Whole-genome sequencing

Positive samples were shipped to the OIE/EU/FAO Reference Laboratory for Avian Influenza, Newcastle Disease and Swine Influenza at the Animal and Plant Health Agency (APHA) in the UK for sequencing. The whole-genome sequence (WGS) data were obtained from four isolates of H5 and seven isolates of H9.

**Data availability statement**

New sequences generated in this study have been submitted to GISAID and can be downloaded using the accession numbers in Table S1. GISAID Acknowledgement table.

### Phylogenetic analysis

The newly generated sequences were combined with all whole-genome sequences available on GISAID, where sampling dates were known and had been sampled from Asian countries. Sequence alignments were curated for H5N1 and H9N2 viruses separately for each gene segment. Maximum likelihood phylogenies reconstructed with RAxML 8.2.11 [18] revealed that the newly generated sequences clustered with previously published Bangladeshi viruses. Molecular clock phylogenies were reconstructed for a subset of the data separately for H5N1 and H9N2 viruses using the Bayesian phylogenetic package BEAST v1.10.4 [19] with a hierarchical phylogenetic model with the following parameters: SRD06 substitution model [20], strict molecular clock, and a Skygrid coalescent prior [21]. For each virus, two independent chains of 50 million steps were executed. The datasets for H5N1 and H9N2 viruses comprised 147 and 95 whole genome sequences, respectively.

### Statistical analyses

We assessed whether co-circulation of H5N1 and H9N2 in a farm was more frequent than expected by chance using a permutation-based test. Permutations were first computed over all farms, regardless of their characteristics. We refer to the number of farms positive to hazard *i*, hazard *j* and both hazards *i* and *j* as $n_{i}$, $n_{j}$ and $n_{ij}$, respectively. In a simulation, we first sampled independently and randomly $n_{i}$ farms and $n_{j}$ farms, and then computed $S_{ij}$, the simulated number of farms positive for both hazards *i* and *j*. A total of 10,000 simulations were conducted, and the proportions of simulations for which $S_{ij}\geq n_{ij}$ and $S_{ij}\leq n_{ij}$ were computed. These corresponded to the *p*-values associated with the observed number of co-infected farms being higher and lower, receptively, than expected by chance. The lowest of the two proportions was reported. In order to assess whether co-infection patterns were influenced by farm characteristics, permutation-based tests were repeated while controlling for the production type (broiler, layer or other), rearing system (deep litter or cages), and the season of the reported outbreak. $N_{kre}$ referred to the number of farms of production system *k*, rearing system *r*, and reporting in season *e*. Among these $N_{kre}$ farms, $n_{krei}$ and $n_{krej}$ were detected as positive for hazards *i* and *j*, respectively. In a simulation, for all possible combinations of farm characteristics {k,r,e}, we sampled $n_{krei}$ farms and $n_{krej}$ farms, and computed $s_{kreij}$, the simulated number of farms of characteristics {k,r,e} positive for both *i* and *j*. The simulated number of co-infected farms was $S_{ij}=\underset{k}{\sum }\underset{r}{\sum }\underset{e}{\sum }s_{kreij}$, and *p*-values were computed as explained above.

We aimed to assess whether the detection of H5 and/or H9 AIV subtypes were associated with a high mortality in the week preceding the report of a disease outbreak in affected chicken flocks. We used logistic regression models with the reported mortality rate in the week preceding sample collection being ≤5% or >5% as the binary outcome variable. Models were built by sequentially introducing sets of variables. The first model included H5 and H9 rRT-PCR results as unique covariates, an interaction term between H5 and H9 rRT-PCR results was added in the second model, and farm characteristics in the third.

## Results

### Occurrence of H5N1 and H9N2 infection in clinically affected chicken flocks

**Figure 1.**
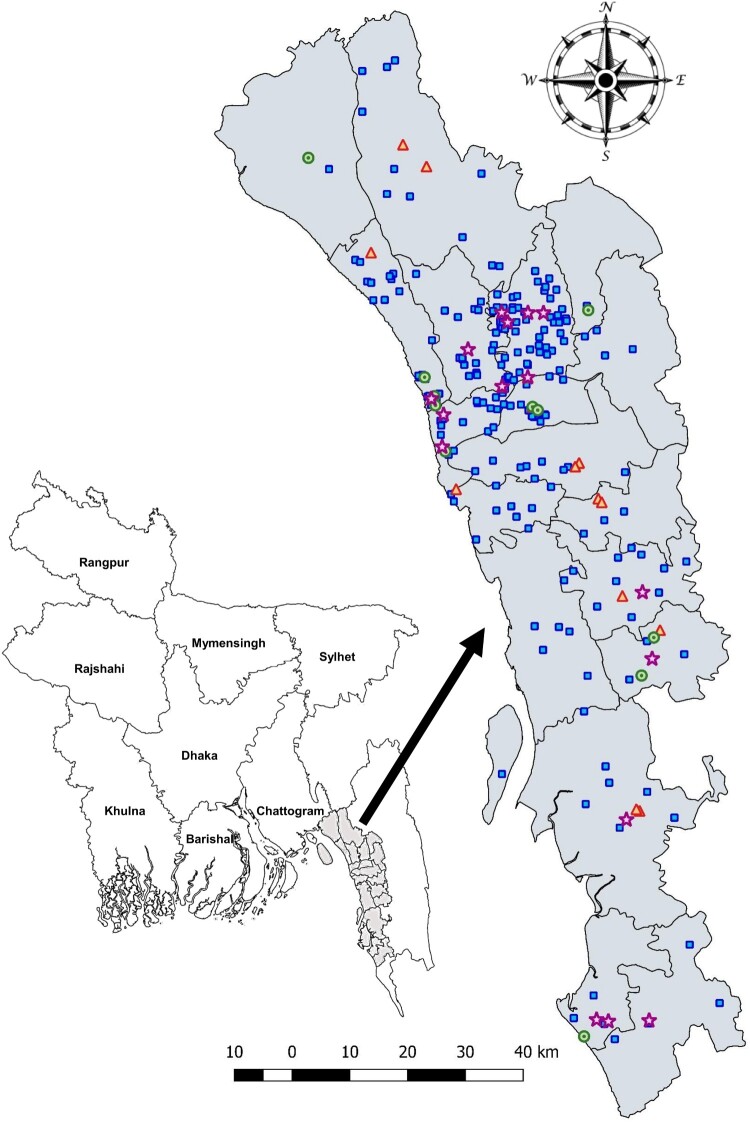
Geographical location of recruited farms. The grey-shaded map shows the sub-districts within Chattogram division where at least one farm was recruited. Farms positive for H5N1 (triangle), H9N2 (star), both subtypes (circle), and negative farms (rectangle) are shown.

Sick and dead chickens were sampled from 262 farms for which veterinary advice was sought. Most of the recruited farms raised exotic broiler chickens (60.3%, *n* = 158), which refers to a fast-growing broiler breed, such as Ross and Cobb 500, or laying hens (29.8%, *n* = 78). Other farms raised either cross-bred sonali broiler chickens (sonali, *Golden* in Bengali, an F1 generation of Fayoumi (♀) and Rhode Island Red (♂)) (8.4%, *n* = 22) or breeders (1.5%, *n* = 4). About a third of farms had 1000–2000 chickens (36.3%, *n* = 95), and 92.4% (*n* = 242) between 500 and 5000 chickens. We set out to test samples by RT-PCR to target the M gene for the identification of influenza-positive samples. We next conducted RT-PCR on influenza-positive samples targeting the HA gene using primers for H5 and H9 (Table S2). We subsequently showed through sequencing that H5-positive isolates were H5N1, and H9-positive isolates were H9N2. Out of 262 farms, 28 (10.7%) and 27 (10.3%) were positive for H5 and H9 viral subtypes, respectively. Twelve were positive for both subtypes. Most were layer farms (58.1%, *n* = 25), with broiler farms accounting for 39.5% (*n* = 17) (Table 1). The study population covered 19 sub-districts, or upazilas, of Chattogram division, and eight thanas of Chattogram city area. RT-PCR positive farms were identified from 14 upazilas and three thanas (Figure 1). The observed number of farms co-infected with H5 and H9 AIV subtypes (*n* = 12) was higher than expected if those infections occurred independently (permutation-based simulations, median: 3 (2.5th–97.5th percentiles: 0–6), *p* = 0), even after controlling for farm characteristics (median: 6 (2.5th–97.5th percentiles: 4–10), *p* = 0.001).

**Table 1.** Number of farms positive for H5 and H9 subtypes.

Variables	All	H5-positive	H9-positive	H5 & H9-positive
*n* (%)	262 (100%)	28 (10.7%)	27 (10.3%)	12 (4.6%)
*Type of production*				
Broiler	158 (60.3%)	7 (4.4%)	16 (10.1%)	6 (3.8%)
Layer	78 (29.8%)	20 (25.6%)	11 (14.1%)	6 (7.7%)
Other	26 (9.9%)	1 (3.8%)	0 (0%)	0 (0%)
*Rearing system*				
Deep litter^a^	219 (83.6%)	12 (5.5%)	19 (8.7%)	8 (3.7%)
Cages	43 (16.4%)	16 (37.2%)	8 (18.6%)	4 (9.3%)
*Flock size*				
<1000	80 (30.5%)	5 (6.2%)	9 (11.2%)	4 (5.0%)
1000–2000	95 (36.3%)	9 (9.5%)	12 (12.6%)	3 (3.2%)
>2000	87 (33.2%)	14 (16.1%)	6 (6.9%)	5 (5.7%)
*Period of disease onset*				
Sep–Nov	100 (38.2%)	2 (2.0%)	5 (5.0%)	1 (1.0%)
Dec–Feb	79 (30.2%)	15 (19.0%)	11 (13.9%)	6 (7.6%)
Mar–May	71 (27.1%)	10 (14.1%)	10 (14.1%)	4 (5.6%)
Jun–Aug	12 (4.6%)	1 (8.3%)	1 (8.3%)	1 (8.3%)

^a^ The whole or only part of the poultry house. Percentages of RT-PCR positive farms is specified with the number of farms for each variable modality (e.g. broiler, layer or other) as the denominator.

Out of the 262 farms recruited in this study, 99 (37.8%) reported a high mortality in the flock, with more than 5% of chickens having died in the week preceding sample collection. The odds of a farm reporting a weekly mortality rate >5% was higher if it was positive for H5 AIVs. There was no evidence of higher odds of reporting high mortality in H9-positive farms, with the odds being reduced if a farm was positive for both H5 and H9 subtypes. The strength of these associations increased when adjusting for farm characteristics, with layer farms having lower odds of reporting high mortality than broiler farms (Table 2).

**Table 2.** Results of the multivariable logistic regression models with the weekly mortality rate as the outcome variable.

Variables	Levels	Weekly mortality		Model 1		Model 2		Model 3	
		<5%	>5%	OR (95% CI)	*p*	OR (95% CI)	*p*	OR (95% CI)	*p*
H5	No	153 (58.4%)	81 (30.9%)	Reference		Reference		Reference	
	Yes	10 (3.8%)	18 (6.9%)	3.14 (1.31–7.52)	0.01	6.00 (1.87–19.25)	0.003	11.26 (2.81–45.18)	0.001
H9	No	150 (57.3%)	85 (32.4%)	Reference		Reference		Reference	
	Yes	13 (5.0%)	14 (5.3%)	1.25 (0.52–3.04)	0.62	2.29 (0.8–6.55)	0.12	2.52 (0.79–8.03)	0.12
Coinfection (H5 and H9)	No	157 (59.9%)	93 (35.5%)			Reference		Reference	
	Yes	6 (2.3%)	6 (2.3%)			0.15 (0.02–0.99)	0.049	0.08 (0.01–0.64)	0.02
Type of chicken production	Broiler	90 (34.4%)	68 (26.0%)					Reference	
	Layer	54 (20.6%)	24 (9.2%)					0.35 (0.15–0.86)	0.02
	Other	19 (7.3%)	7 (2.7%)					0.40 (0.14–1.13)	0.09
Rearing system	Deep litter^a^	135 (51.5%)	84 (32.1%)					Reference	
	Cages	28 (10.7%)	15 (5.7%)					0.71 (0.23–2.20)	0.55
Flock size	<1000	47 (17.9%)	33 (12.6%)					Reference	
	1000–2000	59 (22.5%)	36 (13.7%)					0.84 (0.44–1.60)	0.59
	>2000	57 (21.8%)	30 (11.5%)					1.19 (0.55–2.55)	0.66
Period of disease onset	Sep–Nov	71 (27.1%)	29 (11.1%)					Reference	
	Dec–Feb	39 (14.9%)	40 (15.3%)					2.14 (1.10–4.16)	0.03
	Mar–May	44 (16.8%)	27 (10.3%)					1.32 (0.66–2.63)	0.43
	Jun–Aug	9 (3.4%)	3 (1.1%)					0.92 (0.22–3.79)	0.91

^a^ The whole or only part of the poultry house; OR: odds ratio; *p*: *p*-value.

### Phylogenetic analysis of H5N1 and H9N2 avian influenza viruses

We undertook phylogenetic analysis to ascertain the genetic relationships among the H5N1 and H9N2 viruses detected in the clinically affected farms and their putative closest ancestors. For both H5N1 and H9N2, the sampled viruses clustered closely with other Bangladeshi isolates available on GISAID suggesting that they have emerged from endemic strains circulating in Bangladesh. However, due to limited surveillance in this region, we cannot rule out the possibility of importation outside of Bangladesh. Also, as detailed geographic information for genetically related viruses sampled within Bangladesh was severely limited, we could not determine the spatial dissemination of the sampled viruses in Bangladesh. The H5 HA gene segments all fell within the 2.3.2.1a clade, while the H9 HA gene segments clustered within the G1-W lineage, previously identified as circulating in Bangladesh [10]. As noted by previous studies, despite high levels of co-circulation of H5N1 and H9N2 viruses in the farms, we did not find any evidence for inter-subtype reassortment.

**Figure 2.**
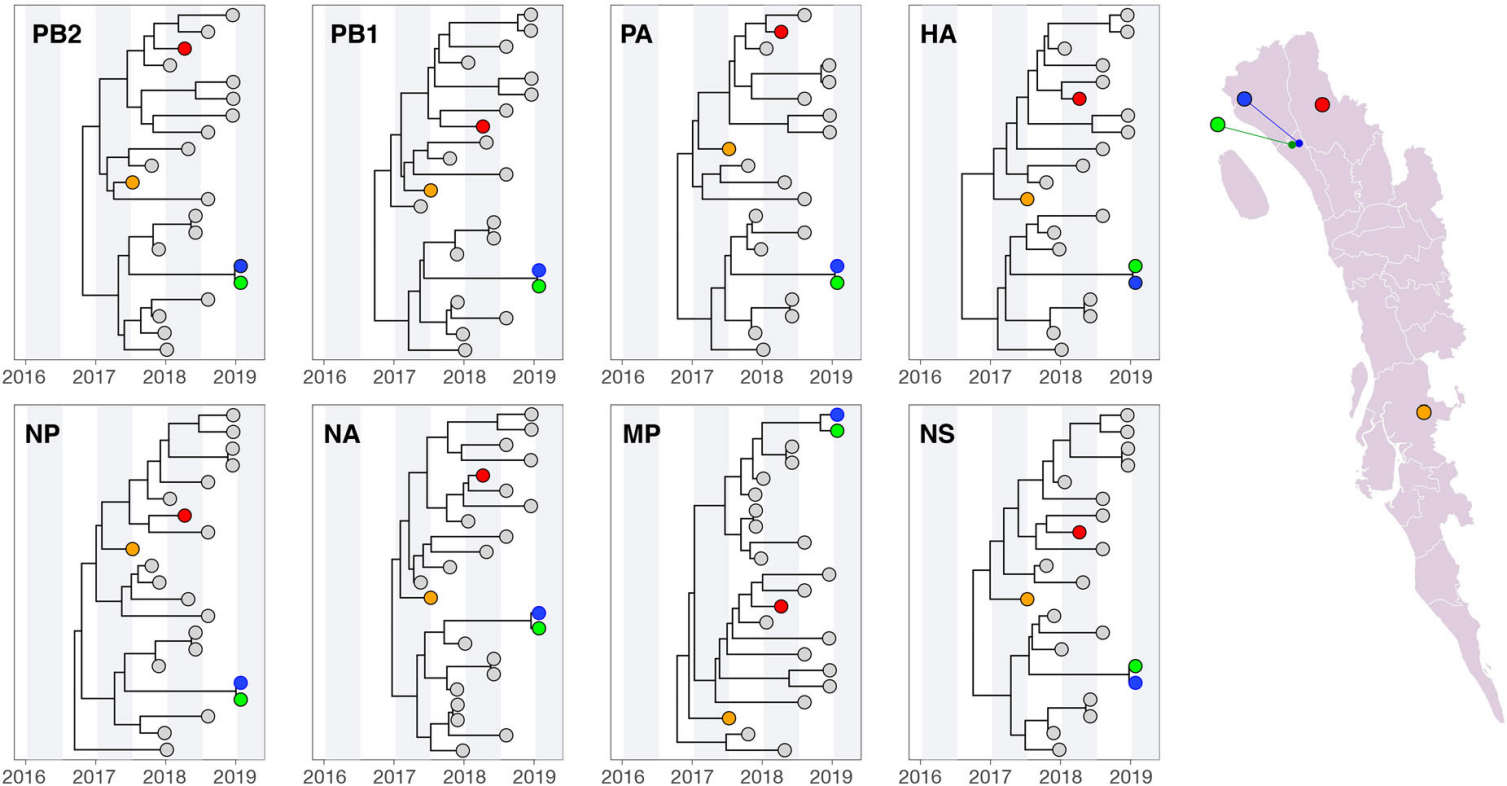
Molecular clock phylogenies of clinical samples of the H5N1 viruses (indicated by coloured circles) isolated from farms (2017–2019). All virus sequences fall within the same subclade in lineage 2.3.2.1.a. Map on the right provide geographic context of where samples were isolated in Chattogram. See Main text and Methods for further details about the analysis.

**Figure 3.**
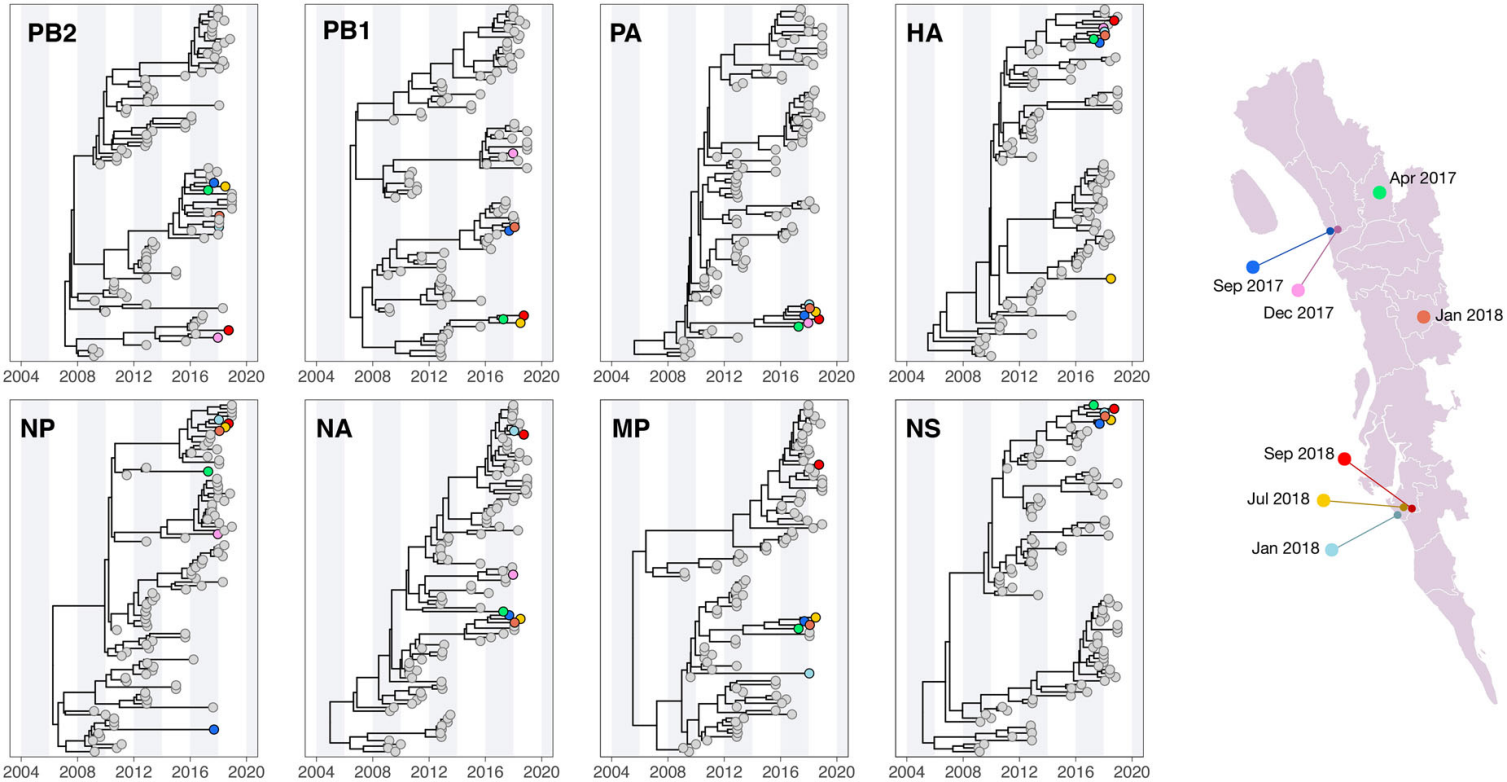
Molecular clock phylogenies of clinical samples of the H9N2 viruses (indicated by coloured circles) isolated from farms (2017–2018). All virus sequences fall within the G1 Western lineage. Map on the right provides geographic context of where and when the samples were isolated in Chattogram. See Main text and Methods for further details about the analysis.

To further understand the emergence of the H5N1 and H9N2 viruses detected in the farms, we reconstructed time-resolved phylogenies for all eight gene sequences from multiple sequence alignments using BEAST v1.10.4 [19] (Figures 2 and 3). In addition to the newly sampled isolates from the farms, we included closely related isolates with whole-genome sequences from GISAID (indicated by grey circles in Figures 2 and 3).

#### H5n1 phylogeny

The H5N1 viruses clustered in the same clade within the 2.3.2.1a lineage in all eight gene segments (Figure 2), which consisted of the same set of virus isolates except for PB1, which included an additional isolate. The average time to the most recent ancestor (TMRCA) of this clade ranged from Aug 2nd, 2016 (95% HPD = Feb 3rd–Nov 28th 2016) to December 20th, 2016 (95% HPD = Aug 31st 2016–Mar 19th 2017), indicating that this clade was circulating in Bangladesh between 2 and 3 years prior to the latest sample detection in early 2019. Furthermore, apart from the two isolates sampled contemporaneously from the two contiguous farms (indicated in blue and green in Figure 2), the H5N1 isolates clustered separately within this clade with high phylogenetic support (posterior probability = 1.0) and non-overlapping node ages (i.e. 95% HPD interval of node ages did not overlap), which suggests that they were introduced independently into this region and correspond to distinct epidemics. However, due to the lack of available metadata, we cannot rule out the possibility that closely related GISAID isolates were also circulating in the same region.

#### H9n2 phylogeny

Figure 3 depicts the evolutionary relationship of the H9N2 viruses sampled from Bangladesh for all eight gene segments. The origins of the seven virus isolate from farms are more complex compared to the H5N1 viruses; the evolutionary histories support substantial reassortment shaping H9N2 virus genetic diversity, and the detected viruses likely emerged from endemic strains that have been co-circulating in Bangladesh for extended periods (between 3 and 9 years). The seven virus isolates correspond to three different clades in HA and four clades in NA (Figure 3). In PB2, NP, and MP, the H9N2 virus isolates fell into multiple distinct clades, while in PA and NS, these isolates clustered into a monophyletic clade with a strong Bayesian statistical support (posterior probability = 1.0; Figure 3). Additionally, these virus isolates did not cluster with a similar group of GISAID isolates in the internal segments, which is consistent with high levels of reassortment.

### Molecular characterization of H5N1 and H9N2 avian influenza viruses

#### Antigenicity

**Figure 4.**
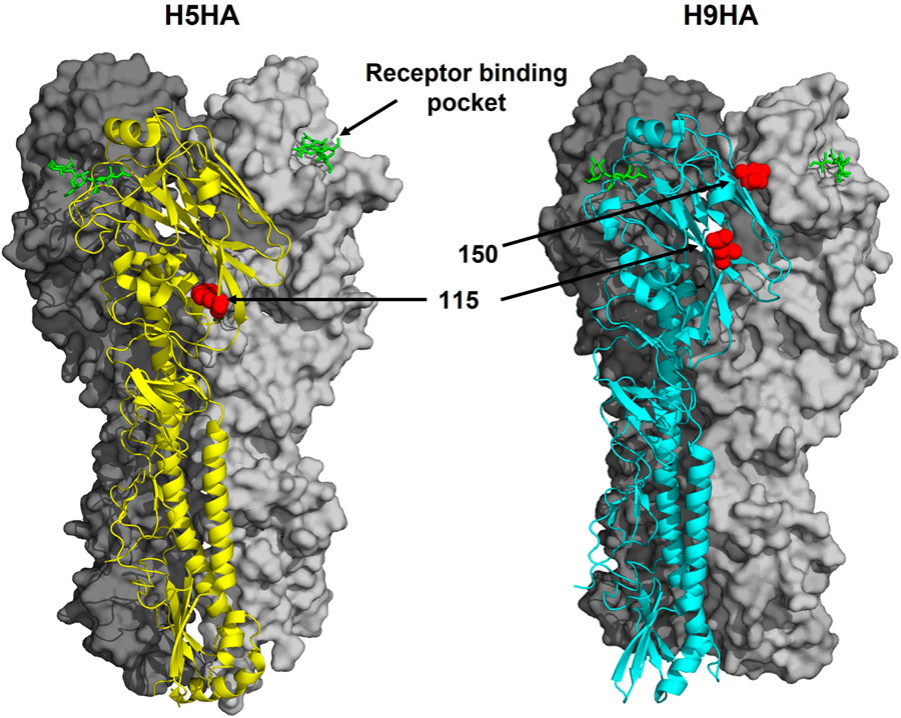
Location of HA amino acid substitutions at antigenic residues in contemporary Bangladesh H5N1 and H9N2 viruses sequenced in this study. Identified residues were mapped onto the crystal structure of H5N1 and H9N2 HA proteins (PDB 5E34 and 1JSH, respectively) [58,59]. HA trimers are shown in cartoon and surface representations with individual amino acid residues represented as red spheres. In green is an LSTa receptor analogue proximal to the receptor binding pocket. Structures were rendered with PyMol [60].

The HA protein is the primary immunogen of influenza viruses, and neutralizing antibodies are directed to this surface glycoprotein during an immune response. As such, mutations in HA can drive antigenic drift, and enable host immune evasion [22]. Analysis of HA amino acid sequences showed that H9N2 viruses detected in the farms were largely homogenous at known antigenic sites; however, a single virus contained L at residue 115 and 150, two antigenic sites, whereas all other viruses contained glutamine (Q) at both these residues. Analysis of antigenic residues in H5N1 viruses sequenced here showed only antigenic residue 115 had any variation (R/Q115) (Figure 4).

To get an appreciation of how Bangladesh H5N1 and H9N2 viruses have evolved historically and in an antigenic context, we analysed our HA sequences alongside previously sequenced H5 and H9 HA genes from Bangladesh (*n* = 305 and 134, respectively). For H9HA there was considerable amino acid diversity at residues 149 and 150, where 25% of viruses after the year 2012, when vaccines were potentially introduced, had drifted from the pre-2012 amino acid species [23]. Namely, G149S and L150F/Q. For residue 149, phylogenetic analysis showed that in some cases, the G149S substitution was continuously maintained for up to 4 years within sub-clades of viruses. Furthermore, the L150F substitution was predominant in quail isolate viruses (*n* = 28/33, 85%). In comparison, only a single chicken isolate contained L150F indicating a role for F150 in H9N2 adaptation of quails (Table S3). For H5HA, nearly complete replacement of amino acids species at residues associated with antigenicity was seen. From the year 2012, residues 53, 115, 120, 129 and 140 saw the replacement of their amino acid species compared to pre-2012. The substitutions R53 K, Q115R, S120D, S129L and R140N first appeared in 2010 in isolates from migratory birds and then subsequently in chicken, duck, quail and crow throughout 2011. However, throughout 2011, there was still variability at each of these residues and it was not until 2012, when vaccines were introduced, that these residues became fixed [24,25] (Table S3). No further changes occurred after this event.

#### Host adaptation, pathogenicity, and drug resistance

Increased fitness of avian influenza viruses to endemic hosts e.g. chickens, or to novel hosts e.g. humans, requires mutations across several viral proteins. Such mutations need to alter virus receptor binding phenotype, pH and thermal stability, and the ability to co-opt host proteins. Therefore, virus adaptation and pathogenicity can involve each viral protein. Analysis of H5N1 and H9N2 sequences highlighted several molecular markers associated with putative host adaptation, pathogenicity, and drug resistance (Tables 3 and 4). Analysis of the cleavage motifs showed all H5N1 viruses contained RRRKR/GLF which is a cleavage motif associated with HPAIVs. H9N2 viruses contained the cleavage motif PAKSKR/GLF, which is a tribasic cleavage motif associated with increased tissue tropism of LPAI viruses in chickens, however, it is currently unclear whether this cleavage site can be associated with enhanced pathogenicity in chickens [26,27]. H5N1 viruses sequenced here contained a single molecular marker for resistance to amantadine and rimantadine in the M2 protein. There was insufficient sequencing coverage of the H9N2 M2 open reading frame to assess molecular markers in the M2 protein. Both H5N1 and H9N2 viruses in Bangladesh carry amino acids markers associated with enhanced adaptation to mammalian hosts, however, two key drivers of mammalian adaptation were absent (Tables 2 and 3). No viruses contained the PB2 E627K amino acid mutation required for polymerase activity in mammalian cells [28], and markers for receptor binding suggest that these H9N2 viruses would preferentially bind sulphated 2,3 linked sialic acid moieties on cell surface receptors, typical of avian viruses. A previous study has assayed the receptor binding phenotype of H9N2 viruses from Bangladesh and found this to be the case [29]. Sequence analysis of the H5N1 viruses also showed that these viruses would likely preferentially bind 2,3 linked sialic acid moieties due to a lack of key markers for human-like 2,6 linked sialic acid binding [30] and lack of human infections with this clade of H5.

**Table 3.** Molecular markers of host adaptation, pathogenicity, and drug resistance in H9N2 viruses.

Virus protein	Phenotype	Reference
*Haemagglutinin*		
Cleavage site PAKSKR	Tribasic residues associated with enhanced tissue tropism in chickens	[26,27]
A29	Adaptation to chickens	[41]
A364	Adaptation to mice	[42]
*Neuraminidase*		
S79	Expanded host range	[41]
N356	Enhanced pathogenicity in mice	[43]
*Polymerase basic 1*		
T39	Adaptation to mice	[44]
A448	Adaptation to mice	[44]
M523	Adaptation to mice	[44]
P607	Adaptation to mice	[44]
*Polymerase acidic*		
S291	Enhanced pathogenicity in mice	[43]
252 aa PA-X (full length)	Virulence factor	[45]
87–90 aa PB1-F2 (full length)	Increase virus shedding in chickens and pathogenesis in mice	[46]
*Nucleoprotein*		
Q231	Adaptation to mice	[44]
T433	Adaptation to mice	[42]
N492	Adaptation to mice	[44]
*Non-structural protein 1*		
K20	Adaptation to mammals	[43]
V180	Adaptation to mammals	[42]

**Table 4.** Molecular markers of host adaptation, pathogenicity, and drug resistance in H5N1 viruses.

Virus protein	Phenotype	Reference
*Haemagglutinin*		
Cleavage site RRRKR	Multibasic residues associated with highly pathogenic avian influenza	[47]
*Neuraminidase*		
49–68 aa stalk deletion	Enhanced pathogenicity in mice	[48]
*Polymerase basic 1*		
G622	Increased polymerase activity in mice	[49]
V3	Increased polymerase activity in avian and mammalian cells	[49]
*Polymerase acidic*		
D383	Increased polymerase activity in avian and mammalian cells	[50]
*Polymerase basic 2*		
R526	Increased polymerase activity in mammalian cells	[51]
N715	Reduced virulence in mice	[51]
*Nucleoprotein*		
V105	Virulence in chickens	[52]
K184	Virulence in chickens	[52]
*Matrix proteins*		
M1 D30	Enhanced virulence	[53,54]
M1 M43	Enhanced virulence	[53–55]
M1 A215	Enhanced virulence	[54,55]
M2 N31	Resistance to amantadine and rimantadine	[54]
*Non-structural protein 1*		
S42	Increased virulence in mice and chickens	[56]
A149	Increased virulence in mice and chickens	[56,57]
PDZ domain motif ESEV	Enhanced virulence in mice	[57]

## Discussion

Our study has characterized the molecular epidemiology of H5N1 and H9N2 viruses in chickens from commercial farms in Bangladesh, which remains a crucial gap in our understanding of AIV transmission in endemically affected regions. Four H5N1 outbreaks in domestic birds in Bangladesh were reported to the OIE in 2017, two in 2018, and none in 2019 [31], suggesting a high level of under-reporting in key poultry populations given the evidence for AIV infections shown here. When facing an outbreak, some farms may not report cases to veterinary authorities due to lack of awareness or concerns about large-scale culling of flocks without any financial compensation, which have long-lasting impacts on their livelihoods [32]. Therefore, HPAI H5 is likely to be more common in commercial farms than suggested by passive surveillance. The co-circulation of H5 and H9 on farms was higher than expected by chance, with the odds of a farm reporting a high mortality rate decreasing in the case of co-infection. If one assumes that viral infections are more likely to be reported if mortality occurs, this could further promote under reporting, leading to significant levels of undetected H5 infections in farming systems. Furthermore, primary infection of chickens with H9N2 can modulate disease severity from secondary infection with H5N1 [33]. There is evidence that H5N1 continues to shed even when morbidity and mortality are reduced, strongly suggesting that H5N1 and H9N2 infections interact in the field. Although vaccines have been reported to be in use in Bangladesh since 2012, there is insufficient data on their precise implementation on farms. Therefore, it is difficult to conclude whether vaccines are having an impact on disease severity reported here. However, if one were to assume that farms reporting H5N1 infection and farms reporting H5N1 and H9N2 co-infection were equally likely to implement H5N1 vaccines then it is indeed likely that H5N1 and H9N2 infections interact in the field to facilitate reduced chicken mortality.

Our findings are somewhat in contrast to the findings from previous surveys in LBMs that reported a higher prevalence of H9N2 relative to H5N1; typically, samples shown to be influenza A positive are subsequently confirmed to be >90% positive for H9N2 compared to only <1% positive for H5 [14,34]. Furthermore, mortality was less severe in the case of H9N2 than H5N1 infection. This is despite the prevalence of H9N2 viruses with a tribasic cleavage motif in the HA protein. The impact of vaccines is also unclear; H5N1 vaccines have been in use in Bangladesh since 2012, however, it has been shown that H5N1 vaccinated chickens remain susceptible to H5N1 infection [23]. Farmers reporting lower mortality in our study might be paying particularly close attention to their flock’s health, whereas most farmers experiencing similar outbreaks may not seek veterinary care. Flocks with mild H9N2 infection would then be less likely to be recruited in our study population. Consequently, the proportion of farms infected by H9N2 likely remains much higher relative to H5N1, as suggested by previous cross-sectional studies [16]. Second, H5N1 transmission from farms to LBMs may be reduced compared to H9N2. While rapid sales of clinically-affected flocks have been reported in Bangladesh [32] as well as in other countries [35], it might not increase H5N1 virus importations in markets if it operates through alternative trading channels, as documented for pigs in Viet Nam [36].

Avian influenza viruses have historically circulated in Bangladesh’s neighbouring countries, highlighting a risk for cross-border transmission [37,38]. However, our findings strongly indicate that H5N1 and H9N2 viruses in Bangladesh repeatedly emerge from endemic lineages. Although the endemic status of H9N2 can be expected, the apparent endemic status of H5 is novel. Therefore, given the endemic status of two AIV subtypes and the propensity for unreported co-infections, it seems likely that genome reassortment could readily occur. We did not detect evidence of recent reassortment between H5N1 and H9N2 viruses; all four H5N1 viruses sequenced in this study corresponded to the same subclade within the 2.3.2.1a lineage. Past reassortment between H9N2 and other HPAIV subtypes was preceded by long-standing evolution of endemic H9N2 viruses that had undergone antigenic drift and become genetically homogenous before successfully reassorting with “exotic” non-endemic HPAIV viruses [39]. However, there seems to be reduced capacity for H5N1 and H9N2 viruses to reassort potentially due to the close relationship of both these viruses with domestic poultry [40].

Analysis of viruses isolated from Bangladesh over the past 15 years suggests H5 and H9 viruses have undergone some degree of antigenic drift. However, it is hard to associate this with vaccine use. For H5 viruses there seemed to be a distinct replacement of amino acids at numerous antigenic residues (53, 115, 120, 129 and 140) since 2012 when H5N1 vaccines were introduced [23]. Antigenic diversity at these residues arose in 2010 likely due to their introduction from migratory birds. Diversity at these residues persisted through to 2011 until 2012 when an apparent fixation event occurred to homogenize the virus population away from the pre-2010 viruses. This event is possibly due to the introduction of H5N1 vaccines. For H9 viruses there was appreciable amino acid diversity at two antigenic residues (149 and 150), however, this diversity persists until today with no fixation event having occurred. If farmers vaccinate against H5N1 and not H9N2 irrespective of flock health, then greater antigenic drift in H5N1 compared to H9N2 could be expected. However, the use of H9N2 vaccines cannot be ruled out due to a lack of information in this area. Indeed, information on in-use vaccine seed strains, regions of use and modalities is scarce. Interestingly, the majority (*n* = 28/33) of quail isolate H9N2 viruses carried the HA substitution L150F. Although residue 150 has been associated with a role in antigenicity, its proximity to the receptor binding pocket and the dearth of F150 in chicken isolate H9N2 viruses could be an indication for this residue being involved quail-specific receptor binding.

Overall, our study highlights multiple LPAI and HPAI outbreaks occurring in Bangladeshi chicken farms, despite limited reporting. We show that H5N1 and H9N2 co-circulate locally, likely emerging from endemic strains circulating in Bangladesh, but are seemingly refractory to reassortment. Their frequent co-occurrence in farms seems to facilitate reduced disease severity, which may contribute to outbreak under-reporting. Finally, we uncover a fixation event at antigenic residues of H5N1 viruses potentially due to the use of H5N1 vaccines.

## Supplementary Material

Table_S3.xlsx

Table_S2.xlsx

Table_S1.xlsx

## References

[1] Biswas PK, Christensen JP, Ahmed SS, et al. Avian influenza outbreaks in chickens, Bangladesh. Emerg Infect Dis.. 2008;14(12):1909. 19046518 10.3201/eid1412.071567 PMC2634614

[2] Gerloff NA, Khan SU, Zanders N, et al. Genetically diverse low pathogenicity avian influenza A virus subtypes co-circulate among poultry in Bangladesh. PLoS One. 2016;11(3):e0152131. 27010791 10.1371/journal.pone.0152131 PMC4806916

[3] Abdelwhab E, Selim A, Arafa A, et al. Circulation of avian influenza H5N1 in live bird markets in Egypt. Avian Dis. 2010;54(2):911–914. 20608538 10.1637/9099-100809-RESNOTE.1

[4] Indriani R, Samaan G, Gultom A, et al. Environmental sampling for avian influenza virus A (H5N1) in live-bird markets, Indonesia. Emerg Infect Dis. 2010;16(12):1889. 21122218 10.3201/eid1612.100402 PMC3294595

[5] Alarcon P, Brouwer A, Venkatesh D, et al. Comparison of 2016–2017 and previous epizootics of highly pathogenic avian influenza H5 Guangdong lineage in Europe. Emerg Infect Dis. 2018;24(12):2270. 30457528 10.3201/eid2412.171860 PMC6256410

[6] Sonnberg S, Webby RJ, Webster RG. Natural history of highly pathogenic avian influenza H5N1. Virus Res. 2013;178(1):63–77. 23735535 10.1016/j.virusres.2013.05.009 PMC3787969

[7] Hassan KE, Ali A, Shany SA, et al. Experimental co-infection of infectious bronchitis and low pathogenic avian influenza H9N2 viruses in commercial broiler chickens. Res Vet Sci. 2017;115:356–362. 28692924 10.1016/j.rvsc.2017.06.024 PMC7172277

[8] Kishida N, Sakoda Y, Eto M, et al. Co-infection of *Staphylococcus aureus* or *Haemophilus paragallinarum* exacerbates H9N2 influenza A virus infection in chickens. Arch Virol. 2004;149(11):2095–2104. 15503199 10.1007/s00705-004-0372-1

[9] Peiris M, Yuen K, Leung C, et al. Human infection with influenza H9N2. Lancet. 1999;354(9182):916–917. 10489954 10.1016/s0140-6736(99)03311-5

[10] Peacock T, James J, Sealy JE, et al. A global perspective on H9N2 avian influenza virus. Viruses. 2019;11(7):620. 10.3390/v11070620 PMC6669617 31284485

[11] Lin YP, Shaw M, Gregory V, et al. Avian-to-human transmission of H9N2 subtype influenza A viruses: relationship between H9N2 and H5N1 human isolates. Proc Natl Acad Sci USA. 2000;97(17):9654–9658. 10920197 10.1073/pnas.160270697 PMC16920

[12] Zhang P, Tang Y, Liu X, et al. A novel genotype H9N2 influenza virus possessing human H5N1 internal genomes has been circulating in poultry in eastern China since 1998. J Virol. 2009;83(17):8428–8438. 19553328 10.1128/JVI.00659-09 PMC2738149

[13] Sealy JE, Fournie G, Trang PH, et al. Poultry trading behaviours in Vietnamese live bird markets as risk factors for avian influenza infection in chickens. Transbound Emerg Dis. 2019;66(6):2507–2516. 31357255 10.1111/tbed.13308 PMC6899644

[14] Kim Y, Biswas PK, Giasuddin M, et al. Prevalence of avian influenza A(H5) and A(H9) viruses in live bird markets, Bangladesh. Emerg Infect Dis. 2018;24(12):2309–2316. 30457545 10.3201/eid2412.180879 PMC6256373

[15] Turner JC, Feeroz MM, Hasan MK, et al. Insight into live bird markets of Bangladesh: an overview of the dynamics of transmission of H5N1 and H9N2 avian influenza viruses. Emerg Microbes Infect. 2017;6(1):1–8. 10.1038/emi.2016.142 PMC5378921 28270655

[16] Gupta SD, Hoque MA, Fournié G, et al. Patterns of Avian Influenza A (H5) and A (H9) virus infection in backyard, commercial broiler and layer chicken farms in Bangladesh. Transbound Emerg Dis. 2020;68(1):137–151. 32639112 10.1111/tbed.13657

[17] Slomka MJ, Pavlidis T, Coward VJ, et al. Validated RealTime reverse transcriptase PCR methods for the diagnosis and pathotyping of Eurasian H7 avian influenza viruses. Influenza Other Respir Viruses. 2009;3(4):151–164. 19627372 10.1111/j.1750-2659.2009.00083.x PMC4634683

[18] Stamatakis A. RAxML version 8: a tool for phylogenetic analysis and post-analysis of large phylogenies. Bioinformatics. 2014;30(9):1312–1313. 24451623 10.1093/bioinformatics/btu033 PMC3998144

[19] Suchard MA, Lemey P, Baele G, et al. Bayesian phylogenetic and phylodynamic data integration using BEAST 1.10. Virus Evol. 2018 Jan;4(1):vey016. 29942656 10.1093/ve/vey016 PMC6007674

[20] Shapiro B, Rambaut A, Drummond AJ. Choosing appropriate substitution models for the phylogenetic analysis of protein-coding sequences. Mol Biol Evol. 2006 Jan;23(1):7–9. 16177232 10.1093/molbev/msj021

[21] Gill MS, Lemey P, Bennett SN, et al. Understanding past population dynamics: Bayesian coalescent-based modeling with covariates. Syst Biol. 2016;65(6):1041–1056. 27368344 10.1093/sysbio/syw050 PMC5066065

[22] Peacock TP, Harvey WT, Sadeyen J-R, et al. The molecular basis of antigenic variation among A(H9N2) avian influenza viruses. Emerg Microbes Infect. 2018;7(1):176. 30401826 10.1038/s41426-018-0178-y PMC6220119

[23] Rimi NA, Hassan M, Chowdhury S, et al. A decade of avian influenza in Bangladesh: where are we now? Trop Med Infect Dis. 2019;4(3):119. 10.3390/tropicalmed4030119 PMC6789720 31514405

[24] Kaverin NV, Rudneva IA, Ilyushina NA, et al. Structure of antigenic sites on the haemagglutinin molecule of H5 avian influenza virus and phenotypic variation of escape mutants. J Gen Virol. 2002;83(10):2497–2505. 12237433 10.1099/0022-1317-83-10-2497

[25] Rudneva IA, Kushch AA, Masalova OV, et al. Antigenic epitopes in the hemagglutinin of Qinghai-type influenza H5N1 virus. Viral Immunol. 2010;23(2):181–187. 20373998 10.1089/vim.2009.0086

[26] Baron J, Tarnow C, Mayoli-Nüssle D, et al. Matriptase, HAT, and TMPRSS2 activate the hemagglutinin of H9N2 influenza A viruses. J Virol. 2013;87(3):1811–1820. 23192872 10.1128/JVI.02320-12 PMC3554176

[27] Parvin R, Schinkoethe J, Grund C, et al. Comparison of pathogenicity of subtype H9 avian influenza wild-type viruses from a wide geographic origin expressing mono-, di-, or tri-basic hemagglutinin cleavage sites. Vet Res. 2020;51:1–12. 32234073 10.1186/s13567-020-00771-3 PMC7106749

[28] Steel J, Lowen AC, Mubareka S, et al. Transmission of influenza virus in a mammalian host is increased by PB2 amino acids 627 K or 627E/701N. PLoS Pathog. 2009;5(1):e1000252. 19119420 10.1371/journal.ppat.1000252 PMC2603332

[29] Peacock TP, Benton DJ, Sadeyen J-R, et al. Variability in H9N2 haemagglutinin receptor-binding preference and the pH of fusion. Emerg Microbes Infect. 2017;6(3):e11. 28325922 10.1038/emi.2016.139 PMC5378918

[30] Rith S, Davis CT, Duong V, et al. Identification of molecular markers associated with alteration of receptor-binding specificity in a novel genotype of highly pathogenic avian influenza A (H5N1) viruses detected in Cambodia in 2013. J Virol. 2014;88(23):13897–13909. 25210193 10.1128/JVI.01887-14 PMC4248976

[31] OIE-WAHIS. Animal disease events: World Organisation for Animal Health; 2021. Available from: https://wahis.oie.int/#/events?viewAll=true.

[32] Høg E, Fournié G, Hoque MA, et al. Competing biosecurity and risk rationalities in the Chittagong poultry commodity chain, Bangladesh. BioSocieties. 2019;14(3):368–392.

[33] Khalenkov A, Perk S, Panshin A, et al. Modulation of the severity of highly pathogenic H5N1 influenza in chickens previously inoculated with Israeli H9N2 influenza viruses. Virology. 2009;383(1):32–38. 18992907 10.1016/j.virol.2008.09.026 PMC2632966

[34] Biswas P, Giasuddin M, Nath B, et al. Biosecurity and circulation of influenza A (H5N1) virus in live-bird markets in Bangladesh, 2012. Transbound Emerg Dis. 2017;64(3):883–891. 26663031 10.1111/tbed.12454

[35] Delabouglise A, Thanh NTL, Xuyen HTA, et al. Poultry farmer response to disease outbreaks in smallholder farming systems in southern Vietnam. Elife. 2020 Aug 25;9:e59212. 32840482 10.7554/eLife.59212 PMC7505654

[36] Hoang MN, Nguyen PT, Han HQ, et al. Network analysis of the sick-pig commodity chain in northern Vietnam: risk of disease dissemination. Revue Délevage Méd Vétér Pays Trop. 2020;73(2):61–70.

[37] Potdar V, Hinge D, Satav A, et al. Laboratory-confirmed avian influenza a (H9N2) virus infection, India, 2019. Emerg Infect Dis. 2019;25(12):2328. 31742537 10.3201/eid2512.190636 PMC6874269

[38] Chowdhury S, Hossain ME, Ghosh PK, et al. The pattern of highly pathogenic avian influenza H5N1 outbreaks in South Asia. Trop Med Infect Dis. 2019;4(4):138. 10.3390/tropicalmed4040138 PMC6958390 31783701

[39] Pu J, Wang S, Yin Y, et al. Evolution of the H9N2 influenza genotype that facilitated the genesis of the novel H7N9 virus. Proc Natl Acad Sci USA. 2015;112(2):548–553. 25548189 10.1073/pnas.1422456112 PMC4299237

[40] Lu L, Lycett SJ, Brown AJL. Reassortment patterns of avian influenza virus internal segments among different subtypes. BMC Evol Biol. 2014;14(1):1–15. 24456010 10.1186/1471-2148-14-16 PMC3905155

[41] Hossain MJ, Hickman D, Perez DR. Evidence of expanded host range and mammalian-associated genetic changes in a duck H9N2 influenza virus following adaptation in quail and chickens. PLoS One. 2008;3(9):e3170. 18779858 10.1371/journal.pone.0003170 PMC2525835

[42] Park KJ, Song M-S, Kim E-H, et al. Molecular characterization of mammalian-adapted Korean-type avian H9N2 virus and evaluation of its virulence in mice. J Microbiol. 2015;53(8):570–577. 26224460 10.1007/s12275-015-5329-4

[43] Zhang Z, Hu S, Li Z, et al. Multiple amino acid substitutions involved in enhanced pathogenicity of LPAI H9N2 in mice. Infect Genet Evol. 2011;11(7):1790–1797. 21896338 10.1016/j.meegid.2011.07.025

[44] Wu R, Zhang H, Yang K, et al. Multiple amino acid substitutions are involved in the adaptation of H9N2 avian influenza virus to mice. Vet Microbiol. 2009;138(1):85–91. 19342184 10.1016/j.vetmic.2009.03.010

[45] Gao H, Liu J, Kong W, et al. PA-X is a virulence factor in avian H9N2 influenza virus. J Gen Virol. 2015;96(9):2587–2594. 26296365 10.1099/jgv.0.000232

[46] James J, Howard W, Iqbal M, et al. Influenza A virus PB1-F2 protein prolongs viral shedding in chickens lengthening the transmission window. J Gen Virol. 2016;97(10):2516–2527. 27558742 10.1099/jgv.0.000584 PMC5078828

[47] Luczo JM, Stambas J, Durr PA, et al. Molecular pathogenesis of H5 highly pathogenic avian influenza: the role of the haemagglutinin cleavage site motif. Rev Med Virol. 2015;25(6):406–430. 26467906 10.1002/rmv.1846 PMC5057330

[48] Matsuoka Y, Swayne DE, Thomas C, et al. Neuraminidase stalk length and additional glycosylation of the hemagglutinin influence the virulence of influenza H5N1 viruses for mice. J Virol. 2009;83(9):4704–4708. 19225004 10.1128/JVI.01987-08 PMC2668507

[49] Feng X, Wang Z, Shi J, et al. Glycine at position 622 in PB1 contributes to the virulence of H5N1 avian influenza virus in mice. J Virol. 2016;90(4):1872–1879. 26656683 10.1128/JVI.02387-15 PMC4733975

[50] Song J, Xu J, Shi J, et al. Synergistic effect of S224P and N383D substitutions in the PA of H5N1 avian influenza virus contributes to mammalian adaptation. Sci Rep. 2015;5:10510. 26000865 10.1038/srep10510 PMC4441148

[51] Song W, Wang P, Mok BW-Y, et al. The K526R substitution in viral protein PB2 enhances the effects of E627K on influenza virus replication. Nat Commun. 2014;5(1):1–12. 10.1038/ncomms6509 PMC4263149 25409547

[52] Tada T, Suzuki K, Sakurai Y, et al. NP body domain and PB2 contribute to increased virulence of H5N1 highly pathogenic avian influenza viruses in chickens. J Virol. 2011;85(4):1834–1846. 21123376 10.1128/JVI.01648-10 PMC3028894

[53] Subbarao K, Klimov A, Katz J, et al. Characterization of an avian influenza A (H5N1) virus isolated from a child with a fatal respiratory illness. Science; 279:393–396. DOI:10.1126/science.279.5349.393. 9430591

[54] Fan S, Deng G, Song J, et al. Two amino acid residues in the matrix protein M1 contribute to the virulence difference of H5N1 avian influenza viruses in mice. Virology. 2009;384(1):28–32. 19117585 10.1016/j.virol.2008.11.044

[55] Nao N, Kajihara M, Manzoor R, et al. A single amino acid in the M1 protein responsible for the different pathogenic potentials of H5N1 highly pathogenic avian influenza virus strains. PLoS One. 2015;10(9):e0137989. 26368015 10.1371/journal.pone.0137989 PMC4569272

[56] Jiao P, Tian G, Li Y, et al. A single-amino-acid substitution in the NS1 protein changes the pathogenicity of H5N1 avian influenza viruses in mice. J Virol. 2008;82(3):1146–1154. 18032512 10.1128/JVI.01698-07 PMC2224464

[57] Li Z, Jiang Y, Jiao P, et al. The NS1 gene contributes to the virulence of H5N1 avian influenza viruses. J Virol. 2006;80(22):11115–11123. 16971424 10.1128/JVI.00993-06 PMC1642184

[58] Ha Y, Stevens DJ, Skehel JJ, et al. X-ray structures of H5 avian and H9 swine influenza virus hemagglutinins bound to avian and human receptor analogs. Proc Natl Acad Sci USA. 2001;98(20):11181–11186. 11562490 10.1073/pnas.201401198 PMC58807

[59] Zhu X, Viswanathan K, Raman R, et al. Structural basis for a switch in receptor binding specificity of two H5N1 hemagglutinin mutants. Cell Rep. 2015;13(8):1683–1691. 26586437 10.1016/j.celrep.2015.10.027 PMC4722862

[60] Schrodinger L. The PyMOL molecular graphics system. Version. 2010;1(5):0.

